# Alopecia Areata

**Published:** 1908-10-17

**Authors:** 


### Dermatology.

### ALOPECIA AREATA.

Alopecia areata, as the name implies, is an affection in which there occur patches or limited areas of baldness upon the scalp. It is a very common affection, and it is met with both in children and in adults. In the majority of cases there are a few bald patches only—from one to five or six—varying in size from that of a sixpenny piece up to a crown or larger. The areas are quite sharply circumscribed, bald, smooth, white, and shining, contrasting with the darker hairy skin around. Towards the margins of the patches there are generally seen a few broken-off stumps of hair which are of normal size and colour at their free end, but atrophied and pale at their attached or root end—the so-called "note of exclamation" hairs.

### Differential Diagnosis.

The affection with which alopecia areata is most often confused is ringworm of the scalp. This confusion need not arise in the case of adults, for they are not subject to ringworm of the scalp. In children, on the other hand, although in typical cases there is a strong contrast between the smooth, white, bald alopecia patch, and the scaly, hair-stump, covered patch of ringworm, yet, under certain conditions, difficulties in diagnosis may arise.

The ringworm patch upon the child's head may be partly bald as the result of inflammation due to some strong application, or the alopecia patch may show an unusual number of "note of exclamation" stumps. But a little care will enable one to distinguish the latter from ringworm stumps, and if the disease is ringworm a careful search should reveal infected stumps which show fungus under the microscope.

### Clinical Course.

The course of alopecia areata is usually very slow, and the patches may remain for many months before they show any sign of regrowth of hair. But after a time fine downy hair appears, which eventually becomes stronger and darker until, finally, all trace of the patch has disappeared. This is what happens in the more favourable cases and, fortunately, in the majority.

But in a certain number of cases the patches gradually extend and coalesce, and fresh ones appear, until the whole of the scalp is entirely bald. It must not be forgotten, too, that alopecia areata frequently involves not only the scalp, but the glabrous skin, the beard, the eyebrows, the eyelashes, the pubes—in fact, any part where there is hair. And in some cases every hair of the body may be lost. Even in these cases, after months or years, the hair may be entirely restored. In many cases of alopecia areata, both in the commoner patchy cases and in the rarer universal cases, the new young hair falls off once or several times before it grows strongly. Relapses after complete regrowth are also not uncommon. In some of the universal cases a permanent regrowth never takes place; and the longer regrowth is delayed the worse is the prognosis.

### Ætiology.

Of the cause of alopecia areata we are quite ignorant. Its clinical features and its histology point rather to a toxic origin than to a microbic one. It is evidently not a disease of the hair itself, like ringworm, but of the hair-papilla. The fall of hair is due, that is to say, not to direct destruction, but to the fact that its nourishment is cut off. The presence of partially-atrophied or "note of exclamation" hairs at the margin of the patch is explained by supposing that the papilla of these hairs have been less severely damaged.

It has recently been suggested by certain observers that the fall of hair in alopecia areata is due to toxins produced by certain microbes which flourish in the mouths of the sebaceous follicles, *e.g.* the micro-bacillus of acne. But this microbe is absent in alopecia areata of children, and it is found after puberty in even quite normal skins, so that it is difficult to accept it as the cause of this affection. Decayed teeth and defective eyesight have also been suggested as exciting causes of alopecia areata. But alopecia areata may occur in persons with perfect teeth and with good eyesight.

### Treatment.

Since we are ignorant of the cause of alopecia our treatment is empirical, and it must be confessed that it is difficult to discover that treatment has any effect in hastening the regrowth of hair or in preventing its further fall. Local stimulation is the usual line of treatment, and a vast number of prescriptions have been invented for this purpose, many of them containing cantharides. More important, perhaps, than local treatment is the connection of any defect in general health. In some cases the administration of intestinal antiseptics has seemed to do good, possibly by removing a causative toxin. In universal cases promotion of sweating by small doses of pilocarpin, and, in limited areas, local injections of pilocarpin ($\frac{1}{10}$ grain) have been suggested. The following are some of the prescriptions recommended for local application:—

℞ Ol. sinapis ... ... ... ... ... ʒj.

Ol. ricini ... ... ... ... ... ʒij.

Sp. rosmarini ... ... ... ... ad ℥iv.

S. To be painted on once or twice a day.

℞ Liq. ammon. fort. ... ... ... ℥ss.

Chloroformi ... ... ... ... ℥ss.

Ol. sesami ... ... ... ... ℥ss.

Ol. limonis ... ... ... ... ʒss.

Sp. rosmarini ... ... ... ... ad ℥iv.

S. To be rubbed gently into the bald part twice a day.

℞ Ung. hydrarg. rubri. ... ... ... ℥j.

Liq. epispasticus ... ... ... ... ♏ xv.

S. The ointment to be rubbed on once a day.

℞ Acet. cantharidis ... ... ... ℥ss.

Hydrarg. perchlor. ... ... ... gr. ij.

Spirit. vini rect. ... ... ... ℥iv.

Ol. lavandulæ ... ... ... ... q.s.

S. The solution to be mopped on once a day.

